# Physicochemical and Functional Characterizations of Biosurfactants Produced by *Pseudomonas aeruginosa* N33 for Oil Removal

**DOI:** 10.3390/microorganisms14010142

**Published:** 2026-01-08

**Authors:** Xinyue Zhao, Meiyu Jiang, Tiantian Du, Xuannuo Liu, Junjia Luo, Yixiang Guo, Xueyu Li, Hongyi Wang, Shiping Wei, Libo Yu

**Affiliations:** 1 School of Marine Sciences, China University of Geosciences, Beijing 100083, China; 2 Key Laboratory of Polar Geology and Marine Mineral Resources (China University of Geosciences, Beijing), Ministry of Education, Beijing 100083, China; 3 Third Institute of Oceanography, Ministry of Natural Resources, Xiamen 361005, China

**Keywords:** *Pseudomonas aeruginosa*, biosurfactant, wettability, emulsification, oil removal

## Abstract

Bacterial biosurfactants have potential applications in green cleaning due to their environmental friendliness. Among all isolated bacterial strains in this study, strain N33 exhibited the most potent oil-displacing activity and was identified as *Pseudomonas aeruginosa*. Its biosurfactant yield was approximately 550 mg/L, and structural characterization revealed it to be a glycolipid-type biosurfactant. The oil-displacing ring diameters of the biosurfactant against vegetable oil, paraffin oil, and crude oil reached 6.3 ± 0.3 cm, 5.8 ± 0.2 cm, and 3.8 ± 0.5 cm, respectively. Its critical micelle concentration (CMC) was determined to be 150 mg/L, with a corresponding surface tension of 39.55 mN/m. Notably, this bacterial biosurfactant significantly improved interfacial wettability, reducing the contact angles of vegetable oil, paraffin oil, and crude oil on oil-wetted glass slides from 93.0°, 99.0°, and 98.8° to 10.0°, 15.0°, and 19.0°, respectively. The emulsification efficiency for the three oils was 80%, 57%, and 10%, respectively. Furthermore, capillary oil removal assays verified that the biosurfactant could efficiently strip oil films from the inner walls of capillaries. These findings demonstrate that the biosurfactant produced by *P. aeruginosa* strain N33 possesses considerable oil-removal efficacy, thereby providing a novel candidate for the research, development, and application of green detergents.

## 1. Introduction

With the rapid advancement of industrialization, oil pollution has emerged as an increasingly prominent issue, becoming a critical challenge that urgently demands attention in the fields of environmental protection and public health [1,2,3]. Oily contaminants, including petroleum hydrocarbons, vegetable oils, and animal fats, originate from various sources, such as industrial manufacturing and food service operations to household cleaning activities and accidental oil spills. These pollutants not only cause severe damage to the ecological environment but also pose long-term and far-reaching threats to water quality, soil fertility, and atmospheric quality [3,4,5,6,7,8]. Consequently, the efficient removal of environmental oil pollution is of significant importance for maintaining healthy ecosystems, ensuring sustainable development, and protecting human health.

Currently, oil contamination cleanup primarily relies on chemically synthesized surfactants such as alkyl sulfonates [4]. Although these surfactants provide benefits in terms of cost-effectiveness, detergency, and chemical stability [9,10], their poor biodegradability and potential environmental toxicity have emerged as critical issues, particularly with the growing demand for eco-friendly products. In contrast, biosurfactants derived from biological sources are favored for their ease of acquisition, high cleaning efficiency, non-toxicity, and low cost, making them a preferred resource in environmental management [3]. Therefore, the development of highly efficient and eco-friendly biodegradable oil-removing detergents has become an inevitable trend [11,12,13,14]. In recent years, bio-based detergents, particularly microbially derived surfactants, have attracted widespread attention due to their high biodegradability, low toxicity, and superior oil-removal capabilities [3,15,16,17,18]. Naturally synthesized through microbial metabolic pathways, these biosurfactants can reduce oil-water interfacial tension, disperse oils into fine droplets, and facilitate emulsification or solubilization, thereby enabling efficient oil removal [14,18,19]. Compared to conventional chemical surfactants, microbially derived detergents offer distinct advantages including rapid action, environmental friendliness, and biocompatibility, rendering them highly versatile across multiple applications [18,20,21,22,23,24]. Beyond household and industrial cleaning, their utility extends to specialized scenarios such as oil spill remediation, heavy oil-contaminated site restoration, and food processing, where they exhibit considerable application potential [3,18].

The ocean, which covers approximately 71% of the Earth’s surface, is characterized by unique environmental conditions including high pressure, low temperature, high salinity, and oligotrophy. These conditions have shaped the distinct metabolic pathways of marine microorganisms, making them a valuable source of novel, high-performance surfactants [25]. In recent years, numerous strains of bacteria capable of producing surfactants have been isolated from diverse marine environments [26,27,28,29]. For instance, Hassanshahian et al. (2014) [27] isolated and screened 14 surfactant-producing bacterial strains from coastal seawater and sediment samples of the Persian Gulf, which belonged to the genera *Shewanella*, *Vibrio*, *Gallaecimonas*, *Brevibacterium*, *Psychrobacter*, and *Pseudomonas*. Among these isolates, *G. pentaromativorans* O15 exhibited the highest surfactant activity, with 10 μL of its fermentation broth achieved a 14 mm oil displacement ring diameter on crude oil. When mixed with hexadecane and left static for 24 h, the emulsification efficiency (E_24_) reached 65%, while the surface tension was reduced to 34.5 mN/m [27]. Sony et al. (2025) [28] isolated 30 bacterial strains from water samples collected from the Chinnamuttom and Muttom harbors (Tamil Nadu, India) and identified 8 strains with robust surfactant-producing activity. Among these isolates, *Stutzerimonas stutzeri* MW15 exhibited high emulsification indices (E_24_) for diesel oil (52.6%), crude oil (51.3%), n-hexadecane (50.5%), n-hexane (48.2%), kerosene (51.8%), and xylene (48.8%). Additionally, this strain exhibited excellent biodegradation performance, achieving an 84% degradation rate for crude oil, making it a promising candidate for oil spill remediation [28]. Zhu et al. (2016) [29] isolated *Bacillus subtilis* N3-4P from petroleum-polluted Atlantic seawater. The biosurfactants produced by this strain reduced the surface tension of water to 27 mN/m and exhibited a critical micellar concentration (CMC) of 507 mg/L. Further investigations demonstrated that these biosurfactants significantly enhanced petroleum removal from contaminated soil: at concentrations ranging from 4 to 8 g/L, the petroleum removal efficiency in soil reached 58–65% [29].

This study focuses on the isolation, screening, and identification of surfactant-producing bacteria from marine sediments. It aims to conduct preliminary structural characterization and performance evaluation of their biosurfactant products, with specific emphasis on critical micellar concentration (CMC), emulsification capacity, wettability properties, and capillary-driven oil removal. The primary objective is to discover high-performance, eco-friendly biosurfactants that can serve as viable alternatives to conventional chemical surfactants. Ultimately, this research aims to establish a robust practical foundation for the removal and remediation of increasingly severe oil contamination.

## 2. Materials and Methods

### 2.1. Isolation of Marine Bacteria

Marine sediment samples were aseptically collected from the Beibu Gulf of the South China Sea, China (109.1437626 °E, 21.40736776 °N). A 10 g sample of marine sediment was added to an Erlenmeyer flask containing 90 mL of distilled water, thoroughly mixed, and allowed to stand for 5 min. The suspension was subjected to 10-fold serial dilution. For each dilution gradient (down to 10^−6^), 50 µL of the diluent was spread onto beef extract-peptone (BEP) medium plates (beef extract 3 g/L, peptone 10 g/L, NaCl 5 g/L, agar 20 g/L, pH 7.2–7.4). The plates were inverted and incubated at 30 °C for 2–3 d until the bacterial colonies appeared, then single colonies were picked for the screening of surfactant-producing strains.

### 2.2. Screening and Identification of Surfactant-Producing Strains

Surfactant-producing bacteria were screened using the oil displacement method [27]. Following the protocol below, single colonies isolated from the plates were inoculated into 5 mL of BEP liquid medium in separate test tubes. The inoculated tubes were incubated at 30 °C with shaking at 150 rpm for 4 d in a constant-temperature shaking incubator (ZQLY-300ES; Shanghai Zhichu Instruments Co., Ltd., Shanghai, China). A 10 cm diameter Petri dish was placed on a coordinate paper, and 30 mL of water was added. Subsequently, 400 µL of Sudan Red-stained paraffin oil was added to the water surface, allowing the oil to spread and form a uniform oil film. After the paraffin oil spread uniformly to form an oil film, 5 µL aliquots of each bacterial fermentation broth were dropped onto the center of the oil film. The formation of oil displacement rings and their diameters were recorded to identify surfactant-producing strains [28,30].

Surfactant-producing bacterial strains were cultured in liquid medium, and their genomic DNA was subsequently extracted. The bacterial 16S rRNA gene was amplified via polymerase chain reaction (PCR) using the universal primers 27F (5′-AGAGTTTGATCCTGGCTCAG-3′) and 1492R (5′-TACCTTGTTACGACTT-3′) [31]. The PCR amplicons were purified and subjected to DNA sequencing. The obtained bacterial 16S rRNA gene sequences were submitted to NCBI database (www.ncbi.nlm.nih.gov, accessed on 26 February 2025) for sequence alignment analysis using BLAST (www.ncbi.nlm.nih.gov, accessed on 26 February 2025). High-similarity sequences from reference strains were selected, and a phylogenetic tree was constructed with MEGA10 software to confirm the taxonomic identification of the isolates.

### 2.3. Extraction of Bacterial Biosurfactants

A single colony of the surfactant-producing bacteria was inoculated into 40 mL of BEP liquid medium and incubated at 30 °C and 150 rpm for 48 h. Subsequently, the culture was inoculated into 1 L of fermentation medium (glucose 5 g/L, peptone 5 g/L, NaNO_3_ 2 g/L, K_2_HPO_4_ 5 g/L, KH_2_PO_4_ 2 g/L, NaCl 0.1 g/L, MgSO_4_·7H_2_O 0.2 g/L, pH 7.0) at a 2% (*v*/*v*) ratio, and incubated at 30 °C with shaking at 150 rpm for 96 h [30]. The culture was centrifuged at 4 °C and 10,000 rpm for 12 min to collect the supernatant. The pH of the supernatant was adjusted to 2.0 using 6 M HCl, and the mixture was kept at 4 °C for 24 h. The mixture was then centrifuged to collect the precipitate, which was washed 2–3 times with acidified water (pH 2.0) until colorless. Finally, the precipitate was freeze-dried under vacuum at −40 °C for 24 h to obtain the bacterial surfactant extract [32]. The extract was weighed to calculate the biosurfactant yield.

### 2.4. Structural Characteristics of Biosurfactants

#### 2.4.1. Determination of Anionic and Cationic Biosurfactants

The extracted bacterial biosurfactants (0.05 g) were dissolved in 100 mL of distilled water to achieve a concentration of 500 mg/L. Meanwhile, solutions of sodium dodecyl sulfate (SDS) and cetyltrimethylammonium bromide (CTAB) at the same concentration were prepared as positive controls for anionic and cationic surfactants, respectively.

The methylene blue method was used for the detection of anionic surfactants [33]. Briefly, 2 mL of the bacterial biosurfactant solution was pipetted into a test tube, followed by the addition of 4 mL of methylene blue solution and 2 mL of chloroform. The mixture was shaken thoroughly and allowed to stand for 10 min. If the chloroform (organic) phase exhibits a noticeable blue color, the bacterial biosurfactant is identified as an anionic surfactant.

For cationic surfactant detection, the bromophenol blue method was adopted [34]. A 4 mL of the bacterial biosurfactant solution was placed in a test tube, and 2–3 drops of bromophenol blue solution were added. The mixture was shaken thoroughly and allowed to stand for 10 min. If the solution in the test tube turns a noticeable blue color, the bacterial biosurfactant is identified as a cationic surfactant.

#### 2.4.2. Determination of Hydrophilic Groups in Bacterial Biosurfactants

A 500 mg/L bacterial biosurfactant solution was prepared. Protein and glucose solutions at the same concentration served as controls, with glucose serving as the positive control for glycosyl groups and protein for amino groups.

The sulfuric acid-anthrone method was used for glycosyl group detection. Briefly, 4 mL of sulfuric acid-anthrone chromogenic reagent (0.1 g of anthrone dissolved in 50 mL of 80% concentrated sulfuric acid, H_2_SO_4_) was added to 1 mL of the bacterial biosurfactant solution. Following thorough mixing, the mixture was incubated in a boiling water bath for 15 min. A blue-green color indicated the presence of glycosyl groups in the bacterial biosurfactant structure [35].

The ninhydrin method was used for amino group detection. Briefly, 1 mL of 2 mol/L acetic acid buffer and 1 mL of ninhydrin chromogenic reagent (0.2 g ninhydrin in 100 mL absolute ethanol) were sequentially added to 2 mL of the bacterial biosurfactant solution. After thorough mixing, the mixture was incubated in a boiling water bath for 15 min. Upon cooling to room temperature, a blue-purple color (Ruhemann’s purple) confirmed the presence of amino groups in the bacterial biosurfactant structure [36].

#### 2.4.3. Fourier Transform Infrared (FTIR) Spectroscopy Analysis

An appropriate amount of the dried bacterial biosurfactant sample was ground into a fine powder using a mortar and pestle. The powdered sample was then mixed with potassium bromide (KBr) powder at a mass ratio of 1:75 [37]. The mixture was loaded into a mold and pressed into a thin pellet. This pellet was then placed in a Fourier transform infrared spectrometer (Nicolet iS10, Thermo Fisher Scientific, Waltham, MA, USA) for spectral scanning. Spectra were recorded over a wavenumber range of 4000–500 cm^−1^ at a resolution of 4 cm^−1^. The resulting FTIR spectra were analyzed to characterize the functional groups of the bacterial biosurfactant [38,39].

### 2.5. Performance Testing of Bacterial Biosurfactants

The oil-removing performance of the bacterial biosurfactant was assessed via its oil displacement activity, critical micellar concentration (CMC), wettability, and emulsification efficiency (E_24_), measured against vegetable oil, paraffin oil, and crude petroleum.

#### 2.5.1. Oil Displacement Activities of Bacterial Biosurfactant Against Different Oils

Oil films were prepared in Petri dishes using 2 mL of vegetable oil (Sudan Red-stained), 2 mL of paraffin oil (Sudan Red-stained), and 2 mL of crude petroleum, respectively. Subsequently, 10 µL of the bacterial biosurfactant solution (500 mg/L) was dropped onto the center of each oil film. The diameter of the oil displacement zone was recorded. The experiment was performed in triplicate.

#### 2.5.2. Determination of CMC of Bacterial Biosurfactant

Bacterial biosurfactant solutions were prepared at serial concentrations ranging from 25 mg/L to 500 mg/L at 25 mg/L intervals. For each concentration, the solution was loaded into a syringe, ensuring no air bubbles were present. The syringe was vertically fixed to the holder of a drop shape analyzer (Model: DSA100, Manufacturer: KRÜSS, Hambury, Germany), and a natural pendant drop (approximately 30 μL) was formed at the needle tip by slowly pushing the plunger. After a 1–2 min stabilization period at 5 °C to ensure constant temperature, lateral images of the symmetrical drop were captured. The Drop Shape Analysis software fitted the drop profile to calculate surface tension and generate a surface tension curve. The CMC was identified as the inflection point of this curve. Each concentration was replicated three times.

#### 2.5.3. Wettability Determination of Bacterial Biosurfactant

Six glass slides were prepared and divided into two groups of three. Each slide was placed in a separate 50 mL centrifuge tube containing vegetable oil, paraffin oil, or crude petroleum, respectively. These tubes were incubated at 80 °C for three days. After incubation, the slides were removed and air-dried to create six oil-wet slides. Three of these oil-wet slides were then immersed in 30 mL of the bacterial biosurfactant solution. The remaining three oil-wet slides were immersed in 30 mL of pure water as the control group. For contact angle measurements, each treated slide was placed on the stage of a contact angle measuring instrument (Drop Shape Analyzer DSA30, KRÜSS). A 5 μL droplet of pure water was dispensed vertically onto the central area of each slide using a microsyringe and allowed to equilibrate for 10–30 s until its shape stabilized. Lateral images of the droplet were captured by the instrument’s camera and imported into the Drop Shape Analysis software to calculate contact angle values.

#### 2.5.4. Determination and Observation of Emulsification Effect of Bacterial Biosurfactant

Emulsification activity was assessed by adding 2 mL of the bacterial biosurfactant solution to separate test tubes containing 4 mL of vegetable oil, paraffin oil, or crude oil. Pure water was used as a control. Thorough emulsification was achieved by vortexing each tube. Following a 24-h incubation period in a dark, constant-temperature environment at 25 °C, the height of the stable emulsion layer (h_emulsion_) was measured. The emulsification efficiency (E_24_) was then calculated using the formula: E_24_ (%) = (h_emulsion_/h_total_) × 100%, where h_total_ refers to the total height of the oil [40]. Emulsion droplets were microscopically observed by placing 20 μL of the emulsion layer on a glass slide and examining it at 400× magnification for size and uniformity.

### 2.6. Capillary Oil Removal Experiment Using Bacterial Biosurfactant

To visualize the oil removal process, glass capillaries were separately filled with vegetable oil (stained with Sudan Red), paraffin oil (also Sudan Red-stained), and crude petroleum [41]. These oil-filled capillaries were incubated at 80 °C for 7 d. Subsequently, 1 mL of deionized water was injected to displace the oil. The inner walls of the capillaries were then observed microscopically to examine the remaining oil films. Finally, the bacterial biosurfactant solution was injected, and the detachment and migration of these oil films from the capillary inner walls were microscopically examined.

## 3. Results

### 3.1. Screening and Identification of Biosurfactant-Producing Bacteria

**Figure 1:**
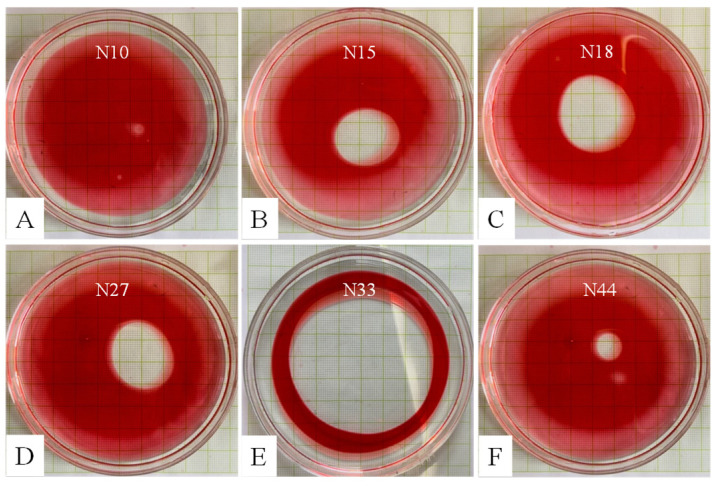
Screening biosurfactant-producing bacteria using the oil displacement method. The paraffin oil was stained with Sudan Red. (**A**), Non-biosurfactant-producing strain; (**B**–**F**), Biosurfactant-producing strains.

A total of 50 morphologically distinct bacterial strains were isolated from marine sediment samples. Each isolated strain was individually inoculated into BEP liquid medium and incubated with shaking for 5 days. A 10 µL aliquot from each culture was then tested for oil displacement activity. The results revealed significant variations in biosurfactant production among the 50 isolated strains. A majority of strains (45 strains, e.g., N10), failed to produce detectable biosurfactants, as evidenced by the absence of noticeable oil displacement rings (Figure 1A). In contrast, five strains (N15, N18, N27, N33, and N44) demonstrated biosurfactant production by forming distinct and clear oil displacement rings (Figure 1B–F). The diameters of the oil displacement rings for these positive strains were measured as 2.5 ± 0.3 cm (N15), 3.1 ± 0.2 cm (N18), 2.5 ± 0.5 cm (N27), 6.0 ± 0.3 cm (N33), and 1.1 ± 0.1 cm (N44). Strain N33 exhibited the largest oil displacement ring diameter (6.0 ± 0.3 cm), indicating superior oil displacement capability and the highest biosurfactant production yield among these five strains.

**Figure 2:**
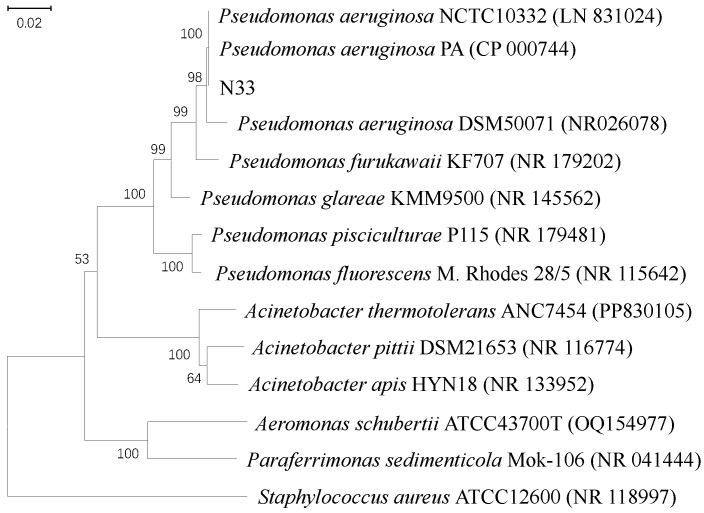
Phylogenetic tree of bacteria N33 with the relatived bacterial strains based on their 16S rRNA gene sequences. Numbers in brackets are GenBank accession numbers. The scale bar represents 2% estimated distance.

The 16S rRNA gene sequence of strain N33 was sequenced and aligned against the GenBank database. The results showed that it shared 99.8% sequence similarity with *Pseudomonas aeruginosa* NCTC10332 (LN831024) and 98.3% similarity with *P. aeruginosa* DSM 50071. A phylogenetic tree was constructed using the 16S rRNA sequences of strain N33 and 13 closely related strains, as shown in (Figure 2). It revealed that strain N33 clustered tightly with *P. aeruginosa* strains, suggesting that strain N33 likely belongs to the species *P. aeruginosa*.

### 3.2. Structural Characterization of Bacterial Biosurfactant

The bacterial surfactant was extracted from the bacterial fermentation broth via acid precipitation, centrifugation, washing, and freeze-drying, yielding 220 mg of crude bacterial surfactant with a production concentration of approximately 550 mg/L. Then, the biosurfactant was performed structurally characterizations.

**Figure 3:**
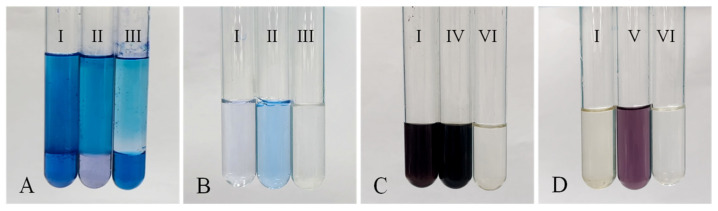
Determination of the anionic and cationic types (**A**,**B**) and hydrophilic group (**C**,**D**) of the bacterial biosurfactant. I, Bacterial biosurfactant; II, CTAB cationic surfactant; III, SDS anionic surfactant; IV, Glucose; V, Protein; VI, Pure water.

The type of bacterial biosurfactants produced by N33 was determined using the methylene blue method and bromophenol blue method, respectively. In the methylene blue assay (Figure 3A), the biosurfactants caused the chloroform layer to turn blue (I in Figure 3), similar to the anionic surfactant SDS (II in Figure 3A). In contrast, in the bromophenol blue assay (Figure 3B), the biosurfactants did not bind to bromophenol blue (I in Figure 3B), and the aqueous phase remained non-blue—this differed from the cationic surfactant CTAB (III in Figure 3B). These results indicate that the biosurfactant produced by strain N33 is an anionic surfactant.

The hydrophilic groups of the biosurfactant produced by strain N33 were determined using the anthrone-sulfuric acid method. The biosurfactant exhibited a distinct color reaction with the sulfuric acid-anthrone solution, turning purple-black (I in Figure 3C), similar to the glucose control group (IV in Figure 3C). In contrast, no color reaction was observed when the biosurfactant reacted with ninhydrin (I in Figure 3D), differing from the protein control group (V in Figure 3D). These results indicated that the hydrophilic group of the bacterial biosurfactant was a glycosyl group rather than an amino group.

**Figure 4:**
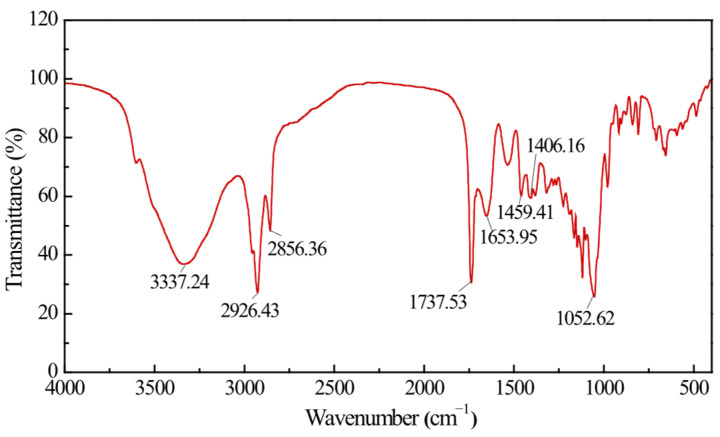
FTIR spectrum of the bacterial biosurfactant.

The FTIR spectrum of the bacterial biosurfactant is shown in Figure 4. The absorption peaks at 3337.24 cm^−1^ and 1052.62 cm^−1^ were attributed to the stretching vibrations of -OH and C-O-C groups, respectively [42], indicating the presence of cyclic carbohydrate structures in the bacterial biosurfactant. The absorption peaks at 2926.43 cm^−1^ and 2856.30 cm^−1^ corresponded to the stretching vibrations of -CH groups, while those at 1459.41 cm^−1^ and 1406.16 cm^−1^ were assigned to the stretching vibrations of -CH_2_ and -CH_3_ groups, respectively. Additionally, the absorption peaks at 1737.53 cm^−1^ and 1653.95 cm^−1^ were characteristic of the C=O stretching vibrations of ester groups and carboxyl groups, respectively [28,38,43], suggesting the presence of fatty acid structures in the bacterial biosurfactant.

Based on the comprehensive analysis of the various assays, the bacterial biosurfactant was determined to be a glycolipid-type anionic biosurfactant.

### 3.3. Performance Evaluation of the Bacterial Biosurfactant

To investigate the oil-displacing performance of the bacterial biosurfactant against different types of oils, vegetable oil, paraffin oil, and crude petroleum were used in this study to investigate its oil-displacing activity, CMC, wettability, and emulsification efficiency, respectively.

**Figure 5:**
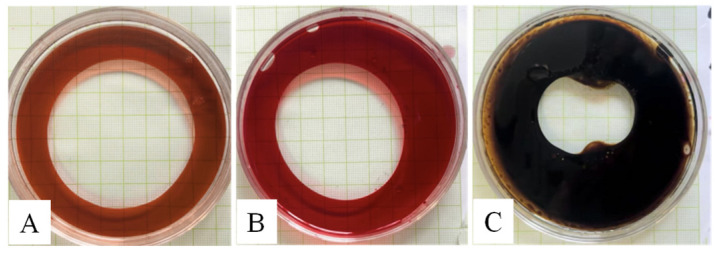
Oil-displacing zones of the bacterial biosurfactant on different oils. (**A**), Sudan Red-stained vegetable oil; (**B**), Sudan Red-stained paraffin oil; (**C**), Crude petroleum.

The oil-displacing activity of the bacterial biosurfactant produced by strain N33 against vegetable oil, paraffin oil, and crude petroleum is shown in Figure 5. The diameters of the oil- displacing rings were determined to be 6.3 ± 0.3 cm, 5.8 ± 0.2 cm, and 3.8 ± 0.5 cm, respectively, with the highest activity observed against vegetable oil.

**Figure 6:**
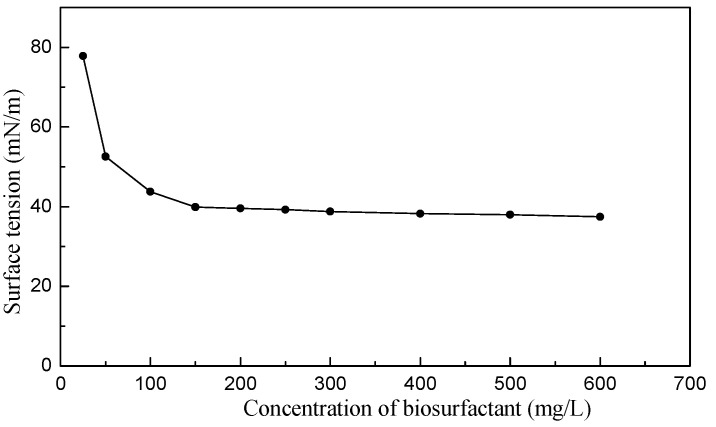
Determination of the CMC of the bacterial biosurfactant.

The CMC of the bacterial biosurfactants was determined, as shown in Figure 6. At low concentrations, the surface tension decreased significantly with increasing biosurfactant concentration due to the adsorption of biosurfactant molecules at the air-water interface. As the biosurfactant concentration increased further, the curve reached an inflection point and plateaued, indicating the formation of micelles and interface saturation. The concentration corresponding to this inflection point was approximately 150 mg/L, which is the CMC of the bacterial biosurfactant, and the surface tension was maintained at around 39.55 mN/m.

**Figure 7:**
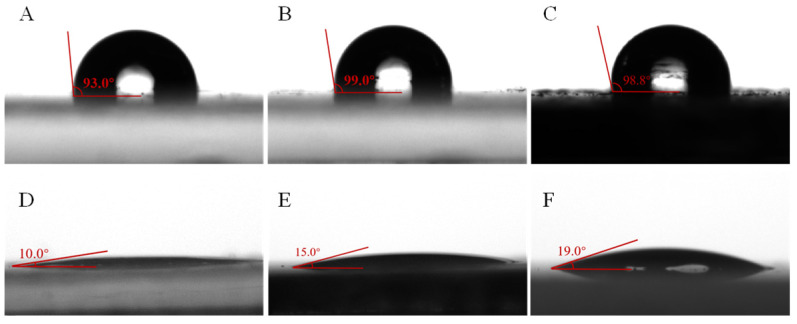
Contact angle measurements. (**A**–**C**) and (**D**–**F**) represent the contact angles of vegetable oil (**A**,**D**), paraffin oil (**B**,**E**), and crude petroleum (**C**,**F**) treated with pure water and the bacterial biosurfactant, respectively.

The wettability of the bacterial biosurfactant was evaluated by measuring the contact angle, as illustrated in Figure 7. The contact angles between oil-coated glass slides (vegetable oil, paraffin oil, and crude petroleum) and pure water were 93.0°, 99.0°, and 98.8°, respectively (Figure 7A–C), all exceeding 90°, indicating hydrophobic surfaces. Consequently, water droplets were difficult to spread and maintained an approximately spherical shape on these surfaces. After treatment with the bacterial biosurfactant, the contact angles decreased significantly to 10.0°, 15.0°, and 19.0°, respectively (Figure 7D–F), demonstrating the transformation of the surfaces to hydrophilic. Water droplets could spread easily and appeared flat on the treated surfaces. The bacterial biosurfactant most effectively modified the wettability of vegetable oil-coated glass slides, followed by paraffin oil-coated and crude petroleum-coated glass slides.

**Figure 8:**
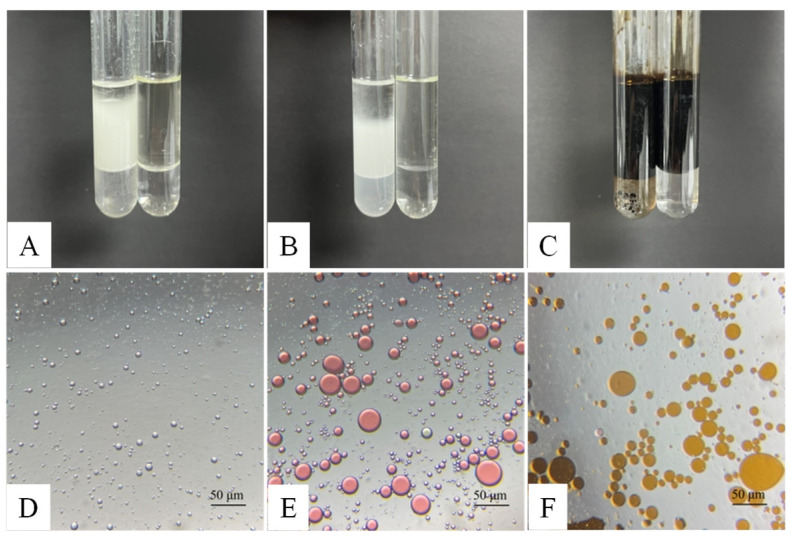
Emulsification effects of the bacterial biosurfactant on vegetable Oil (**A**,**D**), paraffin oil (**B**,**E**), and crude petroleum (**C**,**F**). (**A**–**C**) represent test tube emulsification images; (**D**–**F**) show microscopic observations of emulsion droplets. Sudan Red staining was applied to paraffin oil to facilitate observation.

The emulsification performance of the bacterial biosurfactant against vegetable oil, paraffin oil, and crude petroleum is shown in Figure 8. The bacterial biosurfactant formed a milky white emulsion with vegetable oil, achieving an emulsification efficiency (E_24_) of approximately 80% (Figure 8A). The paraffin oil emulsification system exhibited stratification with moderate stability, with an emulsification efficiency (E_24_) of about 57% (Figure 8B). The emulsification of crude petroleum was confirmed by the decrease in the bottom liquid level, with an emulsification efficiency (E_24_) of approximately 10% (Figure 8C), indicating relatively weak emulsification effectiveness and stability. Microscopic observation of the emulsion droplets (Figure 8D–F) showed that vegetable oil formed densely distributed, uniform droplets (1–8 μm) with minimal large aggregates (Figure 8D), indicative of excellent emulsification performance. In contrast, paraffin oil emulsion droplets showed a broader size distribution (5–20 μm, with some up to 30–40 μm) (Figure 8E), suggesting less uniform emulsification. Crude petroleum emulsion droplets were larger (10–50 μm), discretely and unevenly distributed (Figure 8F), and prone to aggregation, indicating poor emulsification and low system stability. These results demonstrate that the bacterial biosurfactants possess differential emulsification capabilities, performing best with vegetable oil, followed by paraffin oil, and exhibiting the weakest emulsification with crude petroleum.

### 3.4. Capillary Oil Removal Evaluation of the Bacterial Biosurfactant

**Figure 9:**
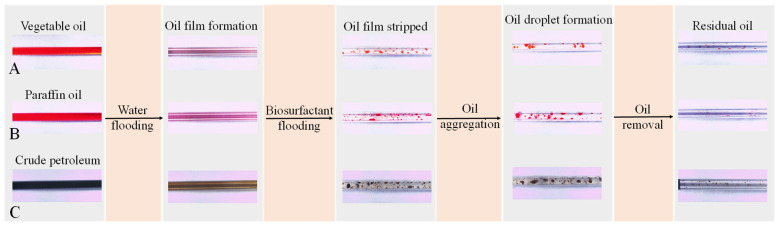
Stereomicroscopic observation (10 × 0.7 magnification) of capillary oil Removal via capillary experiment. (**A**), Vegetable oil; (**B**), Paraffin oil; (**C**), Crude petroleum.

The results of the capillary oil removal experiment are shown in Figure 9. When a capillary filled with vegetable oil was subjected to water flooding, a layer of oil film adhered to the inner wall of the capillary (Figure 9A). Upon injection of the bacterial surfactant, the oil film within the capillary rapidly detached, forming relatively uniform oil droplets that were flushed out. Microscopic observation of the capillary’s inner wall revealed minimal residual oil, indicating a significant oil removal efficiency. The oil removal effect of the bacterial biosurfactant on paraffin oil is illustrated in Figure 9B. Similar to vegetable oil system, after water flooding, a layer of oil film adhered to the inner wall of the paraffin oil-filled capillary (Figure 9B). Following the injection of the bacterial biosurfactant, the paraffin oil film was gradually stripped off, forming dense small-sized oil droplets with high dispersibility. Microscopic observation of the capillary’s inner wall after washing with the bacterial surfactant revealed only a small amount of scattered oil residue, demonstrating a high removal efficiency. The oil removal effect of the bacterial biosurfactant on crude petroleum is shown in Figure 9C. After injecting the bacterial biosurfactant, the oil film adhering to the inner wall of the capillary was effectively stripped off, forming oil droplets that were displaced (Figure 9C). However, microscopic observation revealed partial residual oil on the inner wall. Compared with vegetable oil and paraffin oil, the oil removal effect on crude petroleum was relatively weaker.

## 4. Discussion

### 4.1. Diversity of Biosurfactant-Producing Bacteria

Biosurfactant-producing bacteria are widespread in diverse natural ecosystems, including oil-contaminated areas, industrial wastewater, soil, marine environments, and extreme habitats [29,44,45,46]. The presence and diversity of surfactant-producing bacteria are well-documented across various natural environments. Amani et al. (2010) isolated 102 strains from agricultural and oil-polluted areas in Iran, identifying *Bacillus subtilis*, *P. aeruginosa*, and *Bacillus cereus* as high-efficiency producers [47]. Kaya et al. (2014) isolated two *P. aeruginosa* strains from industrially polluted rivers and oil-contaminated soils in Turkey, both exhibiting high rhamnolipid production [44]. Furthermore, Eldos et al. (2024) identified 11 surfactant-producing bacterial strains from wastewater belonging to the genera *Staphylococcus*, *Corynebacterium*, *Pseudomonas*, *Shewanella*, *Nitratireductor*, *Halomonas*, *Marinobacter*, and *Vibrio* [43]. These findings indicate the extensive presence and variety of surfactant-producing bacteria across different ecosystems. In this study, among 50 bacterial strains isolated from marine sediments, 5 were found to be surfactant-producing, with *P. aeruginosa* N33 exhibiting the highest surfactant production capacity. This aligns with findings by Kubicki et al. (2019), who noted that marine environments are rich sources of diverse surfactant-producing bacteria and biosurfactants [25]. Moreover, while many bacterial taxa produce surfactants, *P. aeruginosa* has become a model strain for current production and a promising candidate for industrial applications due to its potential [48].

### 4.2. Structural Characteristics of Bacterial Surfactants

Bacterial biosurfactants are classified into several major types based on their chemical structures, including glycolipids, lipopeptides, fatty acids, and phospholipids [18,49,50,51,52]. For example, glycolipids encompass rhamnolipids produced by *P. aeruginosa* and *Lisinibacillus sphaericus* [53], sophorolipids by *Starmerella bombicola* and *Torulopsis apicola* [20] and trehalolipids by *Rhodococcus erythropolis* and *Mycobacterium* sp. [54]. Lipopeptides, such as surfactin produced by various *Bacillus* species (e.g., *B. subtilis* and *B. velezensis*), iturin by *B. subtilis*, and lichenysin by *B. licheniformis* [55], are another significant category. Some bacteria, such as *Corynebacterium lepus* and *Thiobacillus thiooxidans*, typically produce fatty acid or phospholipid-type biosurfactants [16]. In this study, preliminary structural identification of the biosurfactant produced by *P. aeruginosa* N33 revealed the presence of glycosyl and carboxyl groups in its structure (Figure 3 and Figure 4). One this basis, the biosurfactant is inferred to belong to the rhamnolipid subclass of glycolipids. According to Eslami et al. (2020), biosurfactants produced by *P. aeruginosa* are generally classified into two mono-rhamnolipids and di-rhamnolipids, which are further subdivided based on differences in their rhamnose moieties and lipid moieties: mono-rhamno-di-lipid, mono-rhamno-mono-lipid, di-rhamno-di-lipid, and di-rhamno-mono-lipid [56]. Although the biosurfactant produced by *P. aeruginosa* N33 was preliminarily identified as an anionic glycolipid-type biosurfactant in this study, its detailed chemical structure requires further characterization.

### 4.3. Properties of Bacterial Surfactants

#### 4.3.1. The CMC of Biosurfactants

The CMC is a crucial parameter for the application of surfactants, as it indicates the minimum concentration required for their efficacy [57]. The CMC of bacterial biosurfactants is strongly correlated with their chemical structures; for example, lipopeptide and glycolipid biosurfactants exhibit significantly different CMC values due to their distinct chemical structures [57,58,59]. Even within the same type of bacterial biosurfactant, variations in CMC may occur. In this study, the CMC of the biosurfactant produced by *P. aeruginosa* N33 was determined to be 150 mg/L, consistent with the findings reported by Kaya et al. (2014) [44]. These authors isolated two *P. aeruginosa* strains from industrial wastewater and oil-polluted soils in Turkey, and the CMC values of their produced rhamnolipids were 115 mg/L and 130 mg/L, respectively [44]. In contrast, Khoshdast et al. (2011) reported a significantly lower CMC of 10.1 mg/L for rhamnolipids produced by a *P. aeruginosa* strain MA01 isolated from spoiled apples [60]. Kłosowska-Chomiczewska et al. (2017) [61] investigated the relationship between the CMC of *P. aeruginosa*-produced rhamnolipid-type biosurfactants and various factors. Their research revealed that the CMC is strongly influenced by purity, pH, and the hydrophobicity of the carbon source substrate. Among these factors, the hydrophobicity of the carbon source had the most significant impact on the CMC of bacterially synthesized rhamnolipids, followed by purity [61].

#### 4.3.2. Biosurfactant Wettability

The wettability of a surfactant refers to its ability to reduce liquid surface tension, facilitating spreading on solid surfaces rather than droplet formation [62]. Surfactants exhibiting superior wettability can swiftly spread and permeate the interstices between oils and solid materials, thus significantly enhancing oil removal effectiveness [63]. Wettability is commonly assessed by contact angle: a contact angle greater than 90° indicates droplet formation and poor wettability (hydrophobic interface), whereas a contact angle less than 90° signifies spreading and good wettability (hydrophilic interface) [64].

In this study, treatment of oil-coated glass slides with vegetable oil, paraffin oil, and crude petroleum with the biosurfactant produced by *P. aeruginosa* N33 resulted in a significant decrease in the contact angle with pure water compared to the control (Figure 7), demonstrating the biosurfactant’s excellent wettability. The contact angle is known to be influenced by the surfactant’s molecular structure and the solid surface properties [65,66,67,68], as well as the chemical composition and polarity of the oil phase. Notably, the contact angles of vegetable oil, paraffin oil, and crude petroleum decreased from 93°, 99°, and 98.8° to 10°, 15°, and 19°, respectively (Figure 7), indicating that variations in wettability are directly linked to the oil’s polarity. Vegetable oil, with its polar ester groups (-COO-), forms weak interactions with the hydrophilic groups of the bacterial biosurfactant. This facilitates biosurfactant adsorption at the oil-water interface, leading to the smallest contact angle. Paraffin oil, consisting mainly of linear alkanes without polar functional groups, exhibits limited interaction with the biosurfactant’s hydrophilic moieties. Consequently, it shows weaker adsorption than vegetable oil, resulting in a contact angle of 15°. In contrast, crude petroleum, a complex mixture of alkanes, naphthenes, aromatics, and heavy components, hinders uniform biosurfactant adsorption at the interface. Consequently, its adsorption efficiency is lower than that of paraffin oil, leading to a contact angle of 19°. These findings indicate that the biosurfactant from *P. aeruginosa* N33 exhibits its strongest wettability against vegetable oil, followed by paraffin oil, and then crude petroleum.

#### 4.3.3. Bacterial Biosurfactant Emulsification

Emulsification fundamentally involves breaking down the dispersed phase (e.g., oil) into microdroplets and ensuring their homogeneous dispersion within the continuous phase (e.g., aqueous). Emulsification efficiency, which reflects the proportion of substances forming a stable emulsion, is influenced by the surfactant’s chemical structure, as well as the viscosity and polarity of the oil phase [28,32]. Rhamnolipids, a glycolipid biosurfactant from *P. aeruginosa* PG1, achieved 100% (E_24_) emulsification efficiency against crude petroleum [69]. Similarly, rhamnolipids from *Stutzerimonas stutzeri* MW15 yielded an E_24_ value of 51.3% for crude petroleum [28]. In contrast, lipopeptide biosurfactants (surfactin and fengycin) from *Cytobacillus* sp. R3-1 showed a crude petroleum emulsification efficiency of 67.37% [70]. Previous studies consistently indicate that glycolipid biosurfactants generally exhibit superior emulsification performance compared to lipopeptides under similar conditions. This is attributed to the highly polar sugar moieties (with multiple hydroxyl groups) in the hydrophilic head of glycolipids, which are more polar than the short peptide chains found in the hydrophilic heads of lipopeptides.

The biosurfactant produced by *P. aeruginosa* N33 showed a notably low E_24_ emulsification efficiency of only 10% for crude petroleum in the present study, which is presumably linked to crude petroleum’s high viscosity. Emulsification efficiency is also influenced by oil phase polarity. As a result, the biosurfactant exhibited significantly higher emulsification efficiencies for vegetable oil and paraffin oil than for crude petroleum (Figure 8A). This difference is likely attributable to variations in oil structure and viscosity, as well as the potentially higher polarity of vegetable oil and paraffin oil compared to crude petroleum. Emulsification efficiency for a given oil phase is typically negatively correlated with the contact angle of the surfactant; a smaller contact angle corresponds to higher emulsification efficiency, and vice versa. This inverse correlation was confirmed by our findings (Figure 7 and Figure 8A). A small contact angle enables oil droplets to more easily disperse into the aqueous phase, where they are effectively encapsulated by water. This prevents droplet reaggregation due to repulsive forces and promotes the formation of stable microdroplets, consistent with the results of this study (Figure 8B). Notably, vegetable oil-coated glass slides, which showed the smallest contact angle, also exhibited the highest emulsification efficiency, smallest droplet diameter, and most uniform droplet distribution, demonstrating superior performance compared to paraffin oil and crude petroleum (Figure 8).

### 4.4. Application of the Bacterial Biosurfactant in Oil Contamination Remediation

Capillary oil removal experiments demonstrated that after water flooding, most of the vegetable oil, paraffin oil, and crude petroleum in the capillaries were flushed out; however, residual oil remained on the inner walls, forming a persistent oil film (Figure 9). Upon adding the biosurfactant produced by *P. aeruginosa* N33, its lipophilic groups inserted into the oil film while the hydrophilic groups oriented outward to bind with water. Additionally, the biosurfactant exhibited excellent wettability, as evidenced by the significant reduction in contact angles (Figure 7), which enhanced water spreading on the glass surface and accelerated the detachment of the oil film from the capillary walls. The detached oil was further dispersed into microdroplets through emulsification and ultimately flushed out with the liquid (Figure 9). These results indicate that the biosurfactant produced by *P. aeruginosa* N33 holds potential as a detergent for the removal of residual vegetable oil, paraffin oil, or petroleum contaminants in the catering industry and industrial settings.

Bacterial biosurfactants show significant promise for applications in catering, household cleaning, and oil contamination remediation due to their low toxicity, biodegradability, and excellent interfacial activity. Kavitha et al. (2014) [71] demonstrated the efficacy of a lipopeptide biosurfactant from the marine bacterium *B. licheniformis* MTCC 5514 in removing crude petroleum from both aqueous and soil phases. In an aqueous phase study, 10% of the biosurfactant achieved an 85% removal efficiency of 2% crude petroleum. In soil remediation, the 10% biosurfactant achieved over 70% removal efficiency for 10% crude petroleum in sandy soil and sandy loam. This performance notably surpassed that of conventional chemical surfactants (SDS, CTAB, Tween 80, and Triton X-100), which achieved less than 60% removal efficiency under identical conditions [71]. Zhu et al. (2016) [29] conducted soil washing experiments on oil-contaminated soil employing a biosurfactant isolated from *B. subtilis* N3-4P, a bacterium sourced from oil-containing seawater in the Atlantic Ocean. Their investigation revealed that crude petroleum removal efficiencies of 58% and 65.2% were achieved at bacterial biosurfactant concentrations of 4 g/L and 8 g/L, respectively [29].

In this study, the assessment of the *P. aeruginosa* N33 biosurfactant’s oil removal efficiency was limited to capillary experiments. Therefore, the quantitative oil removal efficiency of this biosurfactant for vegetable oil, paraffin oil, and crude petroleum requires thorough validation in practical application scenarios.

## 5. Conclusions

In this study, 50 bacterial strains were isolated from marine sediments, screening out 5 surfactant-producing strains, accounting for 10% of the total isolates. This indicates that the marine sediments harbor relatively abundant surfactant-producing bacterial resources. Among these strains, N33 exhibited the highest oil-displacing activity and was identified as *P. aeruginosa*. The biosurfactant produced by this strain reached a yield of 550 mg/L, and preliminary structural characterization classified it as an anionic glycolipid-type biosurfactant. The CMC of this biosurfactant was determined to be 150 mg/L, with a stable surface tension of approximately 39.55 mN/m. It displayed oil-displacing activity against vegetable oil, paraffin oil, and crude petroleum, with the most prominent performance observed for vegetable oil. Additionally, the biosurfactant significantly altered the wettability of solid surfaces, leading to a substantial reduction in the contact angles of different oils. The emulsification rates for vegetable oil, paraffin oil, and crude petroleum were 80%, 57%, and 10%, respectively. Results from capillary oil removal experiments demonstrated that the biosurfactant could rapidly strip off oil films of the three oils, further confirming its efficient oil removal performance. These findings lay a foundation for the application of this biosurfactant in oil removal fields.

## Data Availability

The original contributions presented in this study are included in the article. Further inquiries can be directed to the corresponding authors.
